# Phase transition and structures of the twinned low-temperature phases of (Et_4_N)[ReS_4_]

**DOI:** 10.1107/S205322961901725X

**Published:** 2020-02-07

**Authors:** Eduard Bernhardt, Regine Herbst-Irmer

**Affiliations:** a Anorganische Chemie, Fakultät für Mathematik und Naturwissenschaften, Bergische Universität Wuppertal, Gaussstrasse 20, Wuppertal 42119, Germany; b Institut für Anorganische Chemie, Universität Göttingen, Tammannstrasse 4, Göttingen 37077, Germany

**Keywords:** twinning, phase transition, thio­rhenate, crystal structure

## Abstract

The title com­pound, tetra­ethyl­ammonium tetra­thio­rhenate, [(C_2_H_5_)_4_N][ReS_4_], has, at room temperature, a disordered structure in the space group *P*6_3_ *mc* (*Z* = 2, α-phase). A phase transition to the monoclinic space group *P*2_1_ (*Z* = 2, γ-phase) at 285 K leads to a pseudo-merohedral twin. The high deviation from the hexa­gonal metric causes split reflections. However, the different orientations could not be separated, but were integrated using a large integration box. Rapid cooling to 110–170 K produces a metastable β-phase (*P*6_3_, *Z* = 18) in addition to the γ-phase. All crystals of the β-phase are contaminated with the γ-phase. Additionally, the crystals of the β-phase are merohedrally twinned. In contrast to the α-phase, the β- and γ-phases do not show disorder.

## Abstract 2

The crystal structures of three phases of [(C_2_H_5_)_4_N][ReS_4_] were determined by single-crystal X-ray diffraction analysis at different temperatures.

## Introduction   

Salts with the ReS_4_^−^ anion were synthesized for the first time in 1970 (Müller *et al.*, 1970 ▸). So far, some syntheses of ReS_4_^−^ salts with different cations, such as Me_4_N^+^ (Müller *et al.*, 1970 ▸), Ph_4_P^+^ (Müller *et al.*, 1970 ▸), Ph_4_As^+^ (Müller *et al.*, 1970 ▸; Halbert *et al.*, 1990 ▸; Wei *et al.*, 1991 ▸), Bu_4_N^+^ (Do *et al.*, 1985 ▸), Et_4_N^+^ (Müller *et al.*, 1986 ▸, 1987 ▸; Halbert *et al.*, 1990 ▸; Wei *et al.*, 1991 ▸; Goodman & Rauchfuss, 2002 ▸), Pr_4_N^+^ (Scattergood *et al.*, 1987 ▸) and (PhCH_2_)Et_3_N^+^ (Halbert *et al.*, 1990 ▸; Wei *et al.*, 1991 ▸), have been reported. There is a lack of reliable methods to prepare salts of the ReS_4_^−^ anion with Na^+^, K^+^, Rb^+^ and Cs^+^ cations. The ReS_4_^−^ anion is used in several organic chemistry reactions, such as addition reactions to carbon–carbon multiple bonds (Goodman *et al.*, 1996 ▸; Goodman & Rauchfuss, 1998 ▸, 1999 ▸; Dopke *et al.*, 2000 ▸) and the carbon–nitro­gen triple bond of some nitriles (Goodman & Rauchfuss, 1997 ▸). Moreover, the reaction of the ReS_4_^−^ anion with iso­nitriles has been described (Schwarz & Rauchfuss, 2000 ▸).
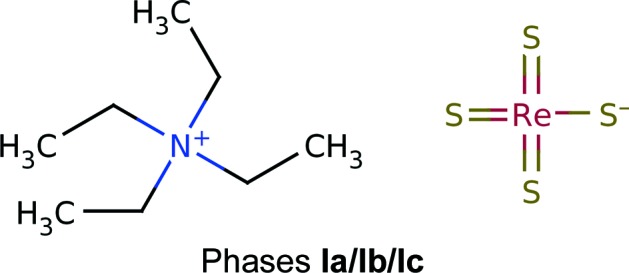


X-ray diffraction studies were published for Ph_4_PReS_4_ (Müller *et al.*, 1970 ▸; Diemann & Müller, 1976 ▸), Ph_4_AsReS_4_ (Müller *et al.*, 1970 ▸), Bu_4_NReS_4_ (Do *et al.*, 1985 ▸) and Et_4_NReS_4_ (Müller *et al.*, 1986 ▸, 1987 ▸). While the structures of Ph_4_PReS_4_, Ph_4_AsReS_4_ and Bu_4_NReS_4_ are ordered, the structure of Et_4_NReS_4_ [*P*6*mm*, *a* = 8.149 (2), *c* = 6.538 (1) Å, *Z* = 1, room temperature] is disordered. Superstructural reflections were observed, suggesting a larger unit cell. The aim of this work was to verify that the unit cell of Et_4_NReS_4_ would be larger at room temperature. Another goal of this work was to investigate whether a phase transition to an ordered structure could be observed at lower temperatures.

## Experimental   

### Synthesis and crystallization   

Et_4_NReS_4_ was synthesized according to the literature method of Goodman & Rauchfuss (2002 ▸). Slow evaporation of an aceto­nitrile solution of Et_4_NReS_4_ in air afforded crystals suitable for X-ray diffraction analysis.

### Refinement   

Crystal data, data collection and structure refinement details are summarized in Table 1 ▸. The phase designations **Ia**–**Ic** are analogous to that used for Et_4_NFeCl_4_ (Lutz *et al.*, 2014 ▸).

#### α-phase   

**Figure 1:**
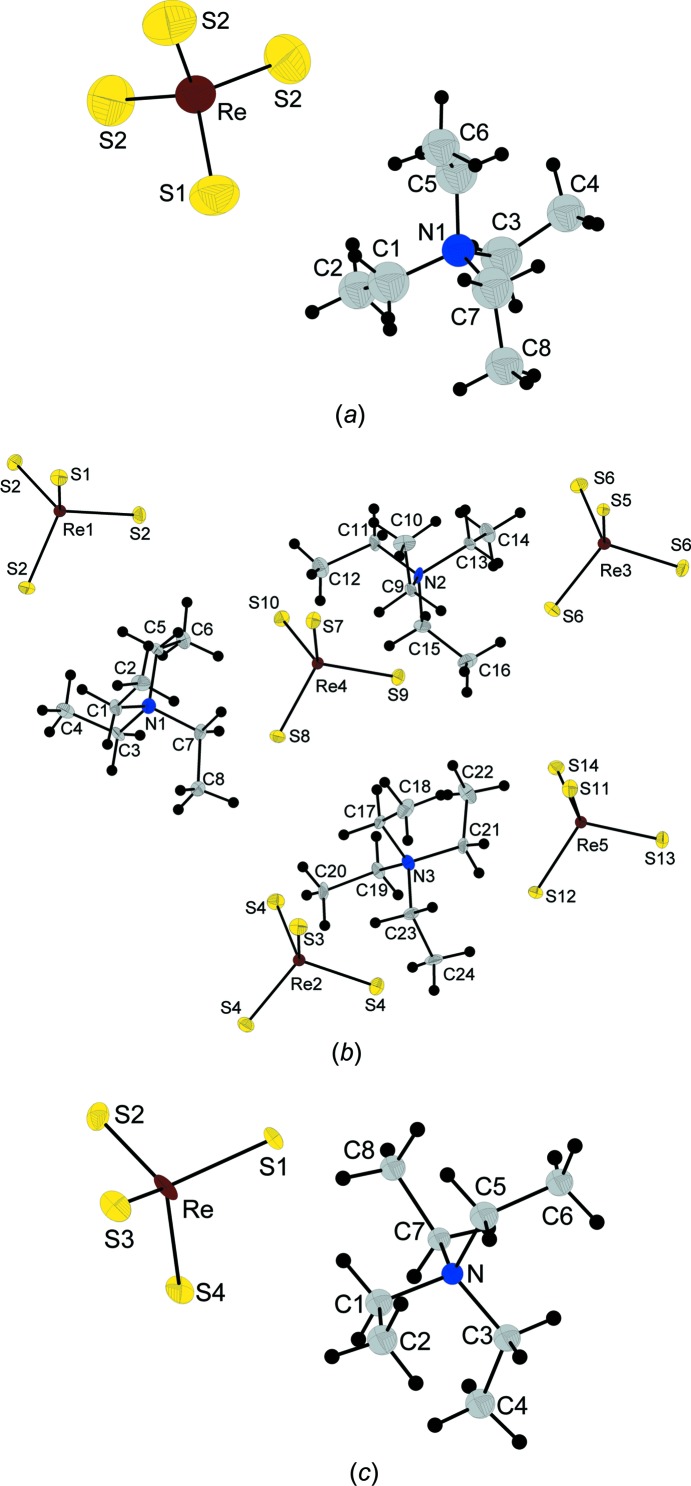
Displacement ellipsoid plots (50% probability level) of (*a*) **Ia** at 297 K, (*b*) **Ic** at 150 K and (*c*) **Ib** at 110 K.

The crystal structure of the α-phase (denoted **Ia**) at 297 K was refined in the space group *P*6_3_ *mc* (Table 1 ▸) starting from the structure of Et_4_NFeCl_4_ at 290 K (Lutz *et al.*, 2014 ▸). The ReS_4_^−^ anion is com­pletely ordered. The whole tetra­ethyl­ammonium cation is disordered about special position *b* with 3*m* symmetry. A whole cation with an occupancy of 1/6 was modelled. Similarity distance restraints were applied for the ethyl groups. All the atoms of the cation were refined isotropically because of this severe disorder, whereas the atoms of the ordered anion were refined anisotropically (Fig. 1 ▸ *a*). H atoms were attached to geometrically optimized positions and refined with the riding model. Twinning by inversion was considered. The fractional contribution of the minor domain refined to 0.14 (4). The C—H distances were fixed at 0.96 (CH_3_) or 0.97 Å (CH_2_). The *U*_iso_(H) values were constrained to 1.5*U*_eq_(C) for methyl H atoms and 1.2*U*_eq_(C) otherwise.

#### γ-phase   

Upon slow cooling (*ca* 5 K min^−1^) to 285 K, crystals of the α-phase (*i.e.* **Ia**) undergo a reversible phase transition to the γ-phase (denoted **Ic**). No further phase transitions could be observed between 110 and 300 K. The γ-phase crystallizes in the space group *P*2_1_ as a pseudomero­hedral twin (Table 1 ▸). Attempts to grow crystals at 273 and 253 K also led to the formation of twins. Data for the γ-phase were collected at 150 K. The high deviation from the hexa­gonal metric leads to split reflections and reflections of different domains close to each other (see Fig. S1 of the supporting information). However, the different orientations could not be separated. To take the twinning into account, an *HKLF5* file (Sevvana *et al.*, 2019 ▸) was produced (*SHELXL2018*; Sheldrick, 2015 ▸) according to the transition from *P*6_3_ *mc* to *P*2_1_ (Table 2 ▸). The normal procedure using the TWIN command was not possible, because in *SHELXL*, only one TWIN command is allowed, but here two twin operations, a threefold and a mirror, are needed. We checked for additional twinning by inversion using now twelve com­ponents, but the fractional contributions of the additional six com­ponents refined to values close to zero (for details, see the supporting information). Both the tetra­thio­perrhenate anion (ReS_4_^−^) and the tetra­ethyl­ammonium cation are com­pletely ordered (Fig. 1 ▸ *b*). However, to stabilize the refinement, distance restraints were used and the cation was only isotropically refined. H atoms were attached to geometrical optimized positions and refined with the riding model. The C—H distances were fixed at 0.98 (CH_3_) or 0.99 Å (CH_2_). The *U*_iso_(H) values were constrained to 1.5*U*_eq_(C) for methyl H atoms and 1.2*U*_eq_(C) otherwise.

#### β-phase   

Rapid cooling (>100 K s^−1^) of the α-phase (*i.e.* **Ia**) to 110–170 K leads to a mixture of the γ-phase (*i.e.* **Ic**) and the β-phase (denoted **Ib**) through a phase transition forming an allotwin. A reciprocal space plot (see Fig. S2 in the supporting information) shows satellites for the reflections with *h* = 3*n* and *k* = 3*m*. With slow heating (*ca* 5 K min^−1^) to 200 K, the β-phase irreversibly changes to the γ-phase. We were not able to obtain crystals of the β-phase free from the γ-phase. Such a superposition of reflections of two phases was also found, for example, in Kautny *et al.* (2017 ▸). The *a* and *b* axes of the β-phase are enlarged by a factor of three com­pared to the γ-phase. Therefore, all *hkl* reflections with *h* = 3*n* and *k* = 3*m* of the β-phase are contaminated with reflections of the γ-phase. The data collection software (*CrysAlis PRO*; Oxford Diffraction, 2016 ▸) could not split the summed intensity into its two com­ponents. Therefore, the *hkl* reflections with *h* = 3*n* and *k* = 3*m* had to be removed from the data set. This lowers the com­pleteness to only 88.8%. Including the con­tami­nated reflections raises the *R*1 value from 0.0354 to 0.0548 and shows *F* ^2^_obs_ values for *hkl* reflections with *h* = 3*n* and *k* = 3*m* much bigger than the *F* ^2^_calc_ values (see Table S1 in the supporting information). Even with the ISOR restraint, where atoms are restrained with effective standard deviations so that their *U* ^*ij*^ components approximate to isotropic behaviour, the anisotropic displacement parameters refine to nonpositive definite values and the residual density increases to 3.45/−3.41 e Å^−3^. The C—H distances were fixed at 0.98 (CH_3_) or 0.99 Å (CH_2_). The *U*_iso_(H) values were constrained to 1.5*U*_eq_(C) for methyl H atoms and 1.2*U*_eq_(C) otherwise.

The β-phase of Et_4_NReS_4_ is isostructural with Et_4_NFeCl_4_ at 230 K (Lutz *et al.*, 2014 ▸). It crystallizes as a merohedral twin with the twin law 010 100 001 and a fractional contribution of 0.5005 (15). This twin law describes a mirror plane perpendicular to the face diagonal. To check for additional twinning by inversion, a refinement with ‘TWIN 0 1 0 1 0 0 0 0 1 −4’ was applied. The additional fractional contributions refined to −0.004 (7) and −0.007 (7). Therefore, twinning by inversion could be excluded. All tetra­thio­perrhenate anions (ReS_4_^−^) and tetra­ethyl­ammonium cations are com­pletely ordered (Fig. 1 ▸ *c*).

## Results and discussion   

At 297 K, Et_4_NReS_4_ is isostructural with Et_4_NFeCl_4_ (Lutz *et al.*, 2014 ▸; Warnke *et al.*, 2010 ▸; Evans *et al.*, 1990 ▸; Navarro *et al.*, 1988 ▸), Et_4_NFeBrCl_3_ (Evans *et al.*, 1990 ▸), Et_4_NInCl_4_ (Trotter *et al.*, 1969 ▸) and Et_4_NTlCl_4_ (Lenck *et al.*, 1991 ▸). They crystallize in the space group *P*6_3_ *mc*. While the anion is ordered, the tetra­ethyl­ammonium cation is disordered. The volume of the primitive cell grows in the following series Et_4_NBF_4_ ≃ Et_4_NClO_4_ < Et_4_NMnO_4_ ≃ Et_4_NPO_2_F_2_ ≃ Et_4_NReOS_3_ < Et_4_NReS_4_ < Et_4_NFeCl_4_ < Et_4_NFeBrCl_3_ < Et_4_NTlCl_4_ ≃ Et_4_NInCl_4_. Accordingly, in the series Et_4_NClO_4_ (378.5 K) > Et_4_NBF_4_ (342 K) > Et_4_NPO_2_F_2_ (323 K) > Et_4_NReS_4_ (285 K) > Et_4_NFeCl_4_ (234.7 K) > Et_4_NTlCl_4_ (222 K), the transition temperature to an ordered structure decreases (Tables 3 ▸ and 4 ▸).

While Et_4_NFeCl_4_ at 234.7 K undergoes a phase transition from *P*6_3_ *mc* to *P*6_3_ (Lutz *et al.*, 2014 ▸; Navarro *et al.*, 1988 ▸), a phase transition from *P*6_3_ *mc* to *P*2_1_ is observed for Et_4_NReS_4_ at 285 K. The *P*6_3_ phase is metastable for Et_4_NReS_4_, while the *P*2_1_ phase is not observed for Et_4_NFeCl_4_. An additional low-temperature phase of Et_4_NFeCl_4_ crystallizes in the space group *Pca*2_1_ [226.6 (1)–2.93 (3) K] (Lutz *et al.*, 2014 ▸; Navarro *et al.*, 1988 ▸). This phase is not observed for Et_4_NReS_4_. While the high-temperature phases of the com­pounds from Table 3 ▸ have the same structure, the low-temperature phases show different structures (Table 4 ▸). Tetra­ethyl­ammonium salts with anions smaller than tetra­thio­rhenate crystallize at room temperature as the low-temperature phase of Et_4_NReS_4_. Whereas the tetra­ethyl­ammonium cation in Et_4_NBF_4_, Et_4_NClO_4_, Et_4_NPO_2_F_2_, Et_4_NReS_4_, Et_4_NFeCl_4_, Et_4_NFeBrCl_3_ and Et_4_NTlCl_4_ has the *tg*–*tg* conformation (*t* is *trans* and *g* is *gauche*), it has the *tt*–*tt* conformation in Et_4_NReO_3_S and Et_4_NMnO_4_ (Naudin *et al.*, 2000 ▸). Only the structures of Et_4_NReO_3_S, Et_4_NMnO_4_, Et_4_NBF_4_ (high-temperature phase) and Et_4_NClO_4_ (high-temperature phase) have an inversion centre. In these two com­pounds, the Et_4_N^+^ cation has a *tg*–*tg* conformation or is disordered.

At 297 K, Et_4_NReS_4_ crystallizes in the space group *P*6_3_ *mc* (Tables 1 ▸ and 4 ▸). Because of the special position of the Re atom (2*a*, 3*m*.; Arnold, 1983 ▸), all reflections with *l* = 2*n* + 1 are much weaker than those with *l* = 2*n* (Table 5 ▸). This could explain why Müller *et al.* (1986 ▸, 1987 ▸) found a smaller primitive cell [*P*6*mm*, *a* = 8.149 (2), *c* = 6.538 (1) Å, *Z* = 1]. They observed weak superstructural reflections, suggesting a doubling of the primitive cell [*a* = 8.149 (2), *c* = 13.076 (2) Å, *Z* = 2], which would be in good agreement with the one found here.

In **Ib** and **Ic**, the Re atom is displaced from the threefold axis. In **Ic**, reflections with *k* = 2*n* + 1 are as strong as the reflections with *k* = 2*n*, while for **Ib** (as also for **Ia**), reflections with *l* = 2*n* + 1 are much weaker than those with *l* = 2*n* (Table 5 ▸).

Structurally, **Ib** is closer to **Ia** than to **Ic**. Therefore, **Ib** is also formed by rapid cooling of **Ia**, although **Ic** is thermodynamically more stable. The energy barrier for the conversion of **Ib** to **Ic** is relatively large, so that rapid conversion occurs only above 200 K.

In the known structures with ReS_4_^−^, the Re—S bond length is independent of the cation (Table 6 ▸). The S—Re—S angle in the ReS_4_^−^ anion is very close to the tetra­hedral value (109.47°). The Re—S bond length in ReO_3_S^−^ is very similar to that in ReS_4_^−^. For ReO_3_S^−^, the following Re—S bond lengths are known: RbReO_3_S with 2.126 (6) Å (Krebs & Kindler, 1969 ▸) and Et_4_NReO_3_S with 2.128 (5) and 2.143 (5) Å (Partyka & Holm, 2004 ▸).

In this article, we were able to show that the unit cell of Et_4_NReS_4_ is larger at room temperature than previously thought (Müller *et al.*, 1986 ▸, 1987 ▸). In this structure, the Et_4_N^+^ cation is disordered, while the ReS_4_^−^ anion is ordered. At 285 K, there is a phase transition to an ordered structure, where the space group changes from *P*6_3_ *mc* to *P*2_1_. The omission of the threefold axis and the mirror plane creates a twin with six com­ponents. In addition to this low-temperature phase, a further metastable phase was formed when **Ia** was cooled rapidly to 110–170 K. This phase crystallizes in the space group *P*6_3_ with a nine times bigger unit cell forming an allotwin with **Ib**.

## Supplementary Material

Crystal structure: contains datablock(s) Ia, Ib, Ic, global. DOI: 10.1107/S205322961901725X/ef3002sup1.cif

Structure factors: contains datablock(s) Ia. DOI: 10.1107/S205322961901725X/ef3002Iasup2.hkl

Structure factors: contains datablock(s) Ib. DOI: 10.1107/S205322961901725X/ef3002Ibsup3.hkl

Structure factors: contains datablock(s) Ic. DOI: 10.1107/S205322961901725X/ef3002Icsup4.hkl

Supporting information file. DOI: 10.1107/S205322961901725X/ef3002Iasup5.cml

Additional figures and table. DOI: 10.1107/S205322961901725X/ef3002sup6.pdf

CCDC references: 1971809, 1971808, 1974401

## Figures and Tables

**Table 1:** Experimental details For all phases: (C_8_H_20_N)[ReS_4_], *M*_r_ = 444.69. Experiments were carried out with Mo *K*α radiation using an Oxford Diffraction Gemini E Ultra diffractometer with an EOS CCD camera. The absorption correction was analytical [*CrysAlis PRO* (Agilent, 2013 ▸), based on expressions derived by Clark & Reid (1995 ▸)]. H-atom parameters were constrained.

	**Ia**	**Ib**	**Ic**
Crystal data			
Crystal system, space group	Hexagonal, *P*6_3_ *m* *c*	Hexagonal, *P*6_3_	Monoclinic, *P*2_1_
Temperature (K)	297	110	150
*a*, *b*, *c* (Å)	8.150 (2), 8.150 (3), 13.092 (3)	24.170 (3), 24.170 (3), 12.916 (2)	7.900 (2), 12.842 (3), 8.118 (2)
α, β, γ (°)	90, 90, 120	90, 90, 120	90, 119.04 (2), 90
*V* (Å^3^)	753.1 (4)	6534.5 (19)	720.0 (3)
*Z*	2	18	2
μ (mm^−1^)	8.59	8.91	8.99
Crystal size (mm)	0.32 × 0.03 × 0.03	0.24 × 0.21 × 0.16	0.24 × 0.20 × 0.17

Data collection			
*T*_min_, *T*_max_	0.599, 0.829	0.253, 0.351	0.241, 0.336
No. of measured, independent and observed [*I* > 2σ(*I*)] reflections	1804, 513, 392	44958, 9707, 8807	4476, 2814, 2808
*R*_int_	0.029	0.036	0.053
(sin θ/λ)_max_ (Å^−1^)	0.632	0.696	0.688

Refinement			
*R*[*F* ^2^ > 2σ(*F* ^2^)], *wR*(*F* ^2^), *S*	0.021, 0.048, 1.08	0.028, 0.048, 1.05	0.052, 0.138, 1.09
No. of reflections	513	9707	2814
No. of parameters	44	380	87
No. of restraints	34	1	89
Δρ_max_, Δρ_min_ (e Å^−3^)	0.26, −0.42	0.87, −1.09	2.01, −4.80
Absolute structure	Refined as an inversion twin	Twinning involves mirror, so Flack parameter cannot be determined	Twinning involves mirror, so Flack parameter cannot be determined
Absolute structure parameter	0.14 (4) (Parsons *et al.*, 2013 ▸)	–	–

Computer programs: *CrysAlis PRO* (Oxford Diffraction, 2016 ▸), *SHELXS97* (Sheldrick, 2008 ▸), *SHELXL2018* (Sheldrick, 2015 ▸) and *DIAMOND* (Brandenburg, 2001 ▸).

**Table 2:** Twin com­ponents in **Ic**

Twin com­ponents	Appropriate symmetry operations in *P*6_3_ *mc*	*h*,*k*,*l*;*i* [*i* = −*h*−*l*]^*^	Fractional contribution *k*_*i*_
1	[1] 1	*h*,*k*,*l*;*i*	0.178 (7)
2	[2] 3^+^ (0,0,*z*)	*l*,*k*,*i*;*h*	0.213 (7)
3	[3] 3^−^ (0,0,*z*)	*i*,*k*,*h*;*l*	0.080 (7)
4	[7] *m (*x*,−*x*,*z*)*	*−l*,*k*,−*h*;*i*	0.084 (7)
5	[8] *m (*x**,2*x,*z*)*	−*h*,*k*,−*i*;*l*	0.233 (7)
6	[9] *m (*2*x,*x*,*z*)*	−*i*,*k*,−*l*;*h*	0.212 (7)

Note: (*) the fourth Miller index is the sum of −*h* and −*l*, because the transformation from *P*6_3_ *mc* to *P*2_1_ causes the 6_3_-axis along the *y* axis.

**Table 3:** Crystallographic data for the high-temperature phase of several Et_4_N*MY*4 com­pounds (*M* = B, Cl, Re, Fe, In and Tl; *Y* = O, F, S, Cl and Br)

Anion	BF_4_^−,*a*^	ClO_4_^−,*b*^	ReS_4_^−,*c*^	FeCl_4_^−,*d*^	FeBrCl_3_^−,*e*^	InCl_4_^−,*f*^	TlCl_4_^−,*g*^
Temperature (K)	373	393	297 (2)	290–295	293	r.t.^*h*^	297
Temperature range (K)	>342	>378.5	>285	>234.7	n.d.^*i*^	n.d.	>222
Space group	*Fm* 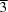 *m*	*Fm* 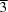 *m*	*P*6_3_ *mc*	*P*6_3_ *mc*	*P*6_3_ *mc*	*P*6_3_ *mc*	*P*6_3_ *mc*
*Z*	4	4	2	2	2	2	2
*V*/*Z* (Å^3^)	317.3 (4)	329.1	376.42 (7)	383.7–385.7	388.4 (1)	397	394.7 (4)

Notes and references: (*a*) Matsumoto *et al.* (2014 ▸); (*b*) Ye *et al.* (2016 ▸); (*c*) this work; (*d*) Lutz *et al.* (2014 ▸), Warnke *et al.* (2010 ▸), Evans *et al.* (1990 ▸) and Navarro *et al.* (1988 ▸); (*e*) Evans *et al.* (1990 ▸); (*f*) Trotter *et al.* (1969 ▸); (*g*) Lenck *et al.* (1991 ▸); (*h*) r.t. = room temperature; (*i*) n.d. = not determined.

**Table 4:** Crystallographic data for the low-temperature phase of several Et_4_N*MY*4 com­pounds (*M* = B, P, Cl, Mn, Re and Fe; *Y* = O, F, S and Cl)

Anion	ClO_4_^−,*a*^	BF_4_^−,*b*^	MnO_4_^−,*c*^	PO_2_F_2_^−,*d*^	ReO_3_S^−,*e*^	ReS_4_^−,*f*^	ReS_4_^−,*f*^	FeCl_4_^−,*g*^	FeCl_4_^−,*h*^
Temperature (K)	110–173	298	293	110	293 (2)	149.9 (3)	109.9 (3)	110–170	230
Temperature range (K)	<378.5	<342	n.d.^*i*^	<323	n.d.	<285	metastable	<226.6	234.7–226.6
Apace group	*Cc*	*Cc*	*P*2_1_/*c*	*Cc*	*P*2_1_/*c*	*P*2_1_	*P*6_3_	*Pca*2_1_	*P*6_3_
*Z*	4	4	4	4	8	2	18	4	18
*V*/*Z* (Å^3^)	291.3–294.5	294.5 (3)	307.7 (1)	311.39 (4)	316.7 (1)	360.0 (2)	363.05 (2)	363.77–367.75	376.86 (3)

Notes and references: (*a*) Ibers (1993 ▸), Kivikoski *et al.* (1995 ▸) and Ye *et al.* (2016 ▸); (*b*) Giuseppetti *et al.* (1994 ▸), Matsumoto *et al.* (2012 ▸) and Matsumoto *et al.* (2014 ▸); (*c*) Whang *et al.* (1991 ▸); (*d*) Matsumoto *et al.* (2012 ▸); (*e*) Partyka & Holm (2004 ▸); (*f*) this work; (*g*) Lutz *et al.* (2014 ▸) and Navarro *et al.* (1988 ▸); (*h*) Lutz *et al.* (2014 ▸) and Navarro *et al.* (1988 ▸); (*i*) n.d. = not determined.

**Table 5:** Intensity of the reflections with even and odd *l* (**Ia** and **Ic**) or *k* (**Ic**) from the .fcf file

	*F* ^2^_av_(odd)/*F* ^2^_av_	*F* ^2^_av_(even)/*F* ^2^_av_	*F* ^2^_av_(odd)/*F* ^2^_av_(even)	Δ^*a*^ (Å)
**Ia** (obs)	0.082	1.737	0.047	0
**Ia** (calc)	0.080	1.740	0.046	0
**Ib** (obs)^*b*^	0.184	1.815	0.102	0.0573^*c*^
**Ib** (calc)	0.174	1.826	0.095	0.0573^*c*^
**Ic** (obs)	0.803	1.196	0.672	0.2720
**Ic** (calc)	0.796	1.203	0.662	0.2720

Notes: (*a*) Average displacement of the Re atoms from the threefold axis. (*b*) Reflections of **Ib** with *h* = 3*n* and *k* = 3*m* overlap with appropriate reflections of **Ic**. The reflections with *h* = 3*n* and *k* = 3*m* are on average 1.2 times too strong and were not used in the refinement. (*c*) Re1, Re2 and Re3 = 0 Å; Re4 = 0.0815 Å; Re5 = 0.0905 Å.

**Table 6:** Re—S bond lengths (Å) and S—Re—S angles (°) for some com­pounds with the ReS_4_^−^ anion

Cation	Et_4_N^+^, **Ib** ^*a*^	Et_4_N^+^, **Ic** ^*a*^	Et_4_N^+^, **Ia** ^*a*^	Et_4_N^+,*b*^	Bu_4_N^+,*c*^	Ph_4_P^+,*d*^
Temperature (K)	109.9 (3)	149.9 (3)	297 (2)	r.t.^*e*^	r.t.	r.t.
Re—S average	2.142 (2)	2.122 (10)	2.125 (4)	2.125 (4)	2.122 (6)	2.155 (30)
Re—S range	2.130–2.154	2.111–2.133	2.120–2.127	2.123–2.126	2.118–2.126	2.155–2.155
S—Re—S average	109.47 (9)	109.47 (71)	109.47 (17)	109.45 (11)	109.48 (84)	Not specified
S—Re—S range	109.06–109.95	108.01–110.61	109.33–109.61	109.4–109.5	107.4–112.8	Not specified

Notes and references: (*a*) this work; (*b*) Müller *et al.* (1987 ▸); (*c*) Do *et al.* (1985 ▸); (*d*) Diemann & Müller (1976 ▸); (*e*) r.t. = room temperature.
