# Supplementary material for: Phase transition and structures of the twinned low-temperature phases of (Et4N)[ReS4]
Source: Acta Crystallogr C Struct Chem. 2020 Feb 7;76(Pt 3):231–5. doi: 10.1107/S205322961901725X (PMC7057183; doi:10.1107/S205322961901725X)
Supplement: Supplementary file 6 [file c-76-00231-sup6.pdf]

STRUCTURAL CHEMISTRY

Volume 76 (2020)

Supporting information for article:

Phase transition and structures of the twinned low-temperature phases of $(Et_4N)[ReS_4]$

Eduard Bernhardt and Regine Herbst-Irmer

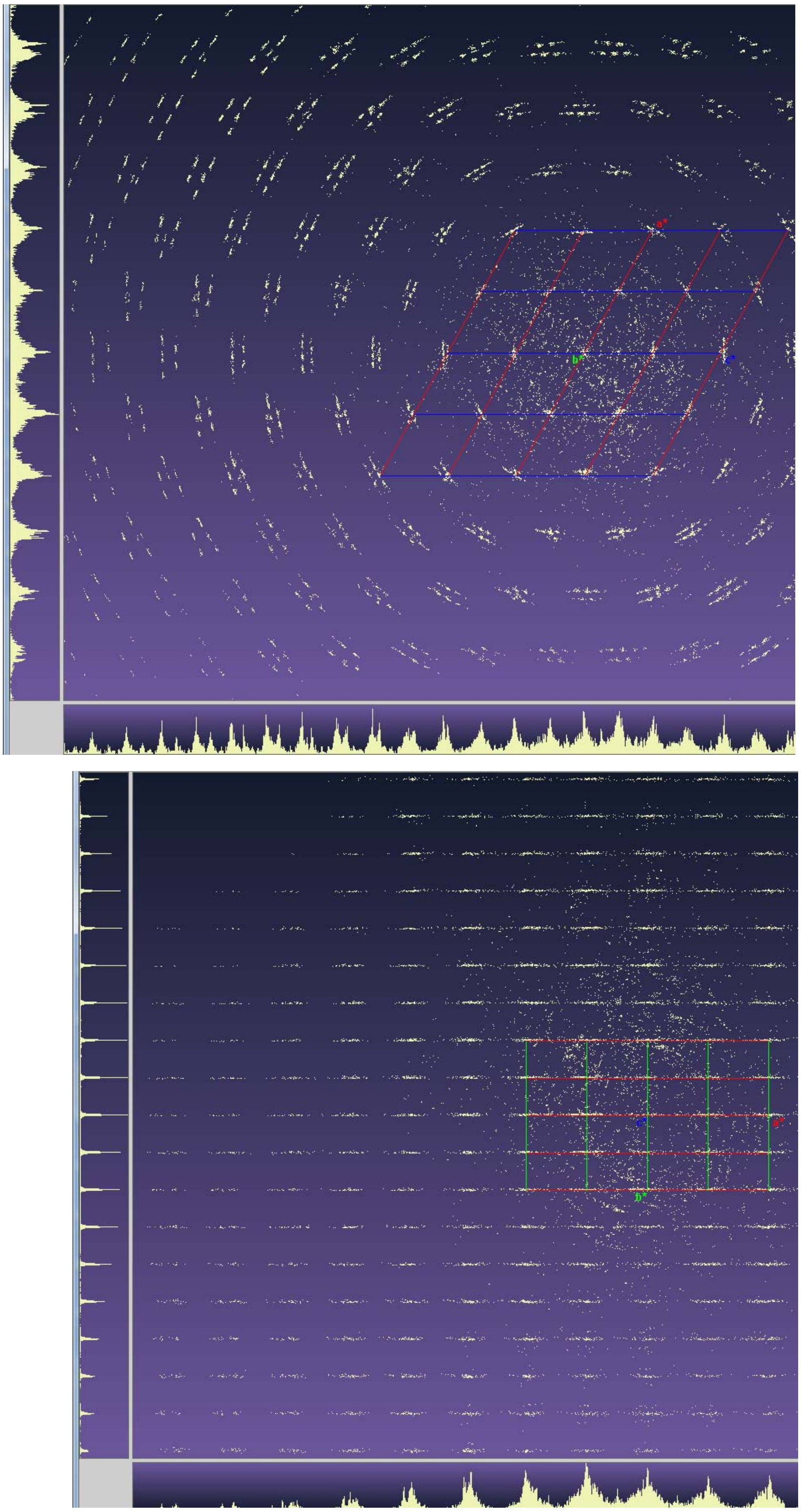

Figure S1 Reciprocal space plot for **Ic** (along b* axis) and (along c* axis). Ewald Explorer (1.0.5) (*CrysAlisPRO*, 2016)

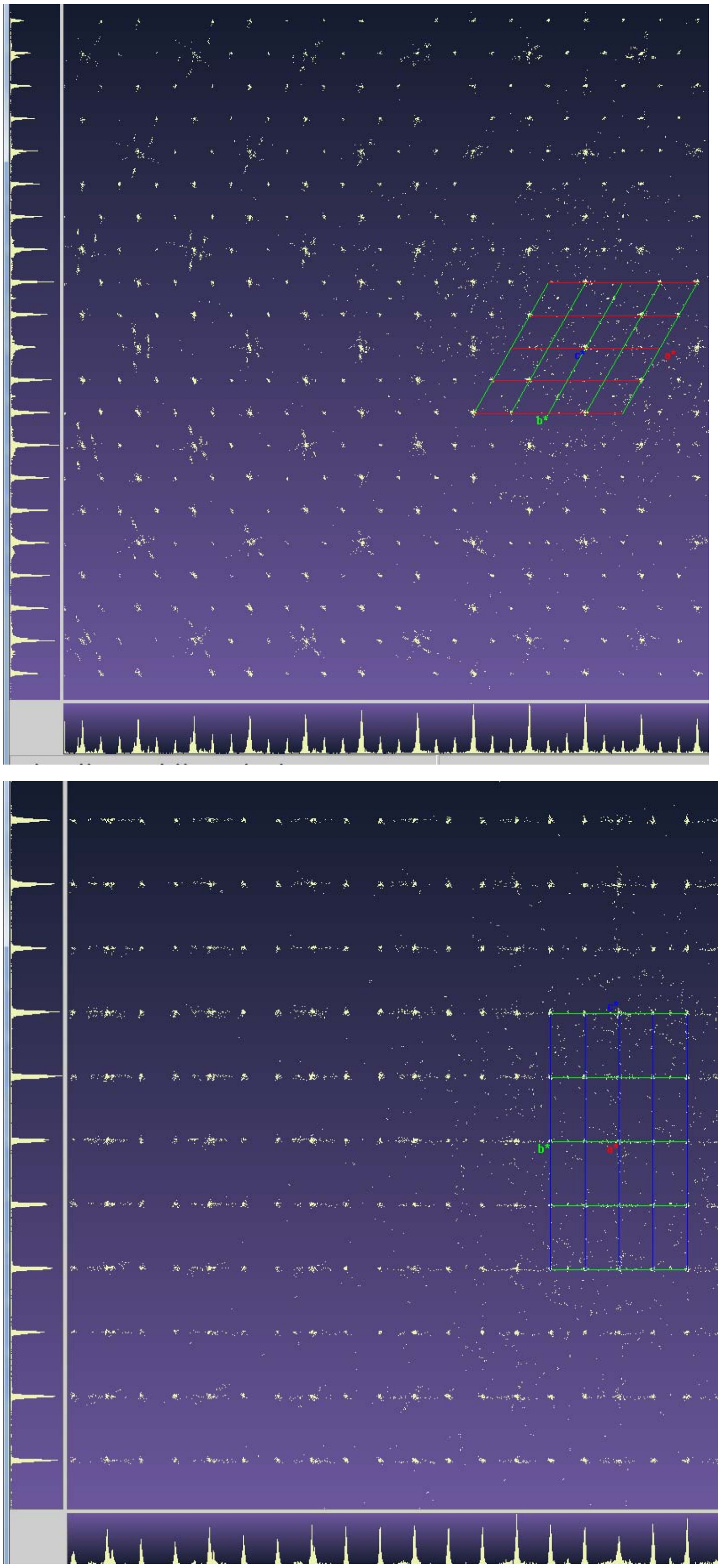

Figure S2 Reciprocal space plot for **1b** (along c* axis) and (along a* axis) Ewald Explorer (1.0.5) (*CrysAlisPRO*, 2016)

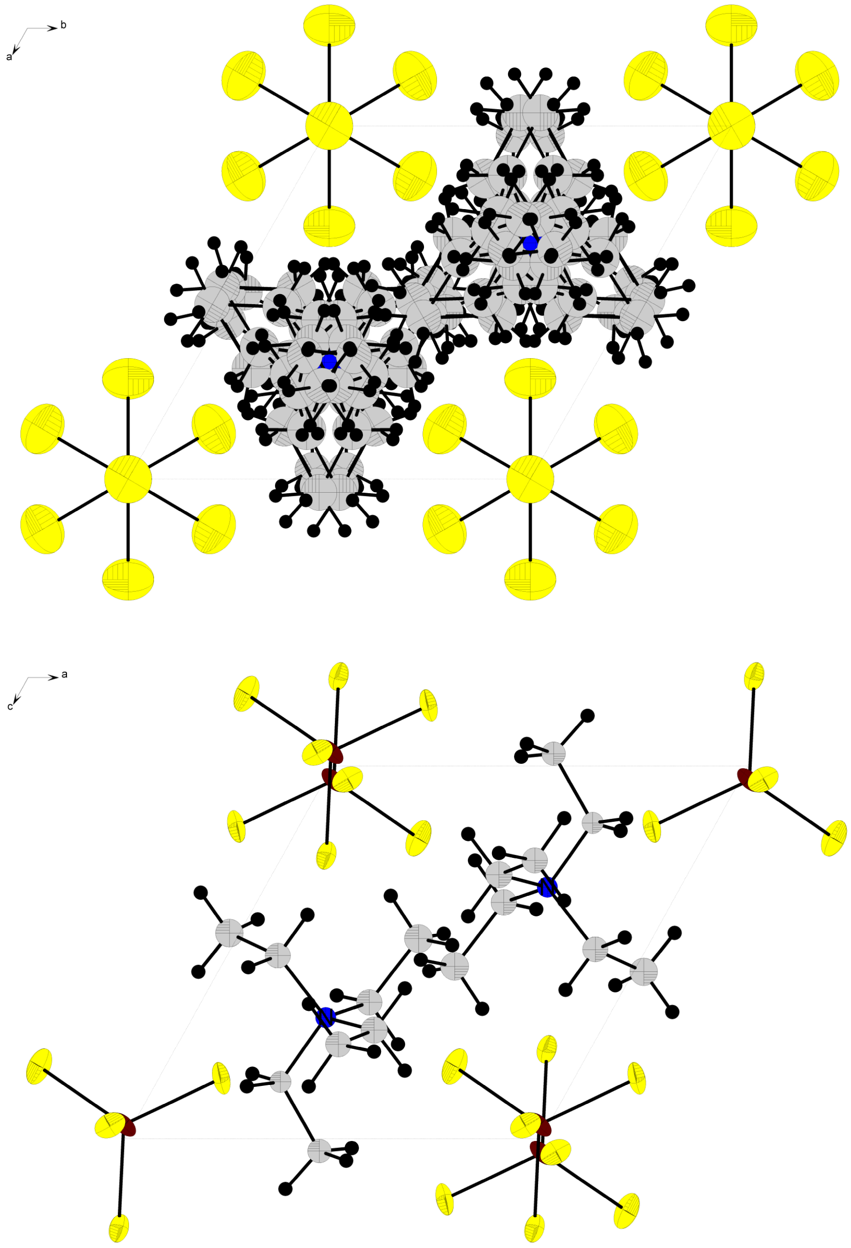

Figure S3 Displacement ellipsoid plot the unit cell of **Ia** at 297 K and **Ic** at 150 K (50% probability level).

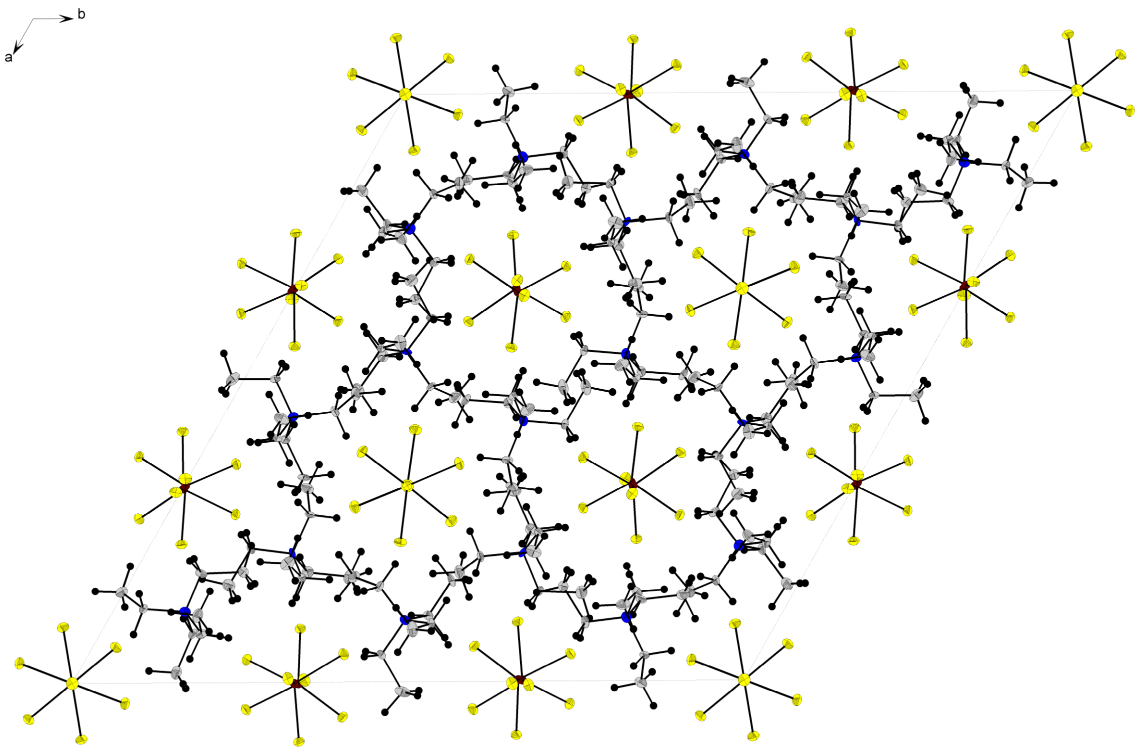

Figure S4 Displacement ellipsoid plot the unit cell of Ib at 110 K (50% probability level).

Table S1. Twin components in **Ic**

Twin compo-nents	*h,k,l;i* * *i* = -*h*-*l*	12 component	6 component	sum from 12 component refinement
1 (1)	*h,k,l;i*	0.14(3)	0.178(7)	0.18 {1+7}
2 (2)	*l,k,i;h*	0.18(3)	0.213(7)	0.22{2+8}
3 (3)	*i,k,h;l*	0.06(3)	0.080(7)	0.08 {3+9}
4	*-l,-k,-h;-i*	0.00(3)		
5	*-h,-k,-i;-l*	0.05(3)		
6	*-i,-k,-l;-h*	0.04(3)		
7	*-h,-k,-l;-i*	0.04(3)		
8	*-l,-k,-i;-h*	0.04(3)		
9	*-i,-k,-h;-i*	0.02(3)		
10 (4)	*-l,k,-h;i*	0.08(3)	0.084(7)	0.08{4+10}
11 (5)	*-h,k,-i;l*	0.19(3)	0.233(7)	0.24 {5+11}
12 (6)	*-i,k,-l;h*	0.16(3)	0.212(7)	0.20 {6+12}

* The fourth Miller index is the sum of -h and -l, because the transformation from $P6_3mc$ to $P2_1$ causes the 6_3-axis along the *y*-axis
